# *Coxiella burnetii* lipopolysaccharide blocks p38α-MAPK activation through the disruption of TLR-2 and TLR-4 association

**DOI:** 10.3389/fcimb.2014.00182

**Published:** 2015-01-06

**Authors:** Filippo Conti, Nicolas Boucherit, Veronica Baldassarre, Virginie Trouplin, Rudolf Toman, Giovanna Mottola, Jean-Louis Mege, Eric Ghigo

**Affiliations:** ^1^CNRS UMR 7278, IRD198, INSERM U1095, Aix-Marseille UniversitéMarseille, France; ^2^Laboratory for Diagnosis and Prevention of Rickettsial and Chlamydial Infections, Institute of Virology, Slovak Academy of SciencesBratislava, Slovakia; ^3^UMR MD2, Faculté de Médecine NORD, Aix-Marseille Université and IRBA (Institute of Research in Biology of the French Army)Marseille, France; ^4^Laboratory of Biochemistry, La Timone University Hospital, Assistance Publique Hôpitaux de MarseilleMarseille, France

**Keywords:** TLR-2, TLR-4, cytoskeleton, *Coxiella burnetii*, macrophages

## Abstract

To survive in macrophages, *Coxiella burnetii* hijacks the activation pathway of macrophages. Recently, we have demonstrated that *C. burnetii*, via its lipopolysaccharide (LPS), avoids the activation of p38α-MAPK through an antagonistic engagement of Toll-like receptor (TLR)-4. We investigated the fine-tuned mechanism leading to the absence of activation of the p38α-MAPK despite TLR-4 engagement. In macrophages challenged with LPS from the avirulent variants of *C. burnetii*, TLR-4 and TLR-2 co-immunoprecipitated. This association was absent in cells challenged by the LPS of pathogenic *C. burnetii*. The disruption makes TLRs unable to signal during the recognition of the LPS of pathogenic *C. burnetii*. The disruption of TLR-2 and TLR-4 was induced by the re-organization of the macrophage cytoskeleton by *C. burnetii* LPS. Interestingly, blocking the actin cytoskeleton re-organization relieved the disruption of the association TLR-2/TLR-4 by pathogenic *C. burnetii* and rescued the p38α-MAPK activation by *C. burnetii*. We elucidated an unexpected mechanism allowing pathogenic *C. burnetii* to avoid macrophage activation by the disruption of the TLR-2 and TLR-4 association.

## Introduction

*Coxiella burnetii* is an intracellular bacteria responsible of the Q fever zoonosis and is a potential bio warfare and bioterrorism agent (Regis, 1999; Madariaga et al., 2003). Q fever is characterized by a lethal endocarditis (Raoult et al., 2005). It has been shown that molecular variations in *C. burnetii* lipopolysaccharide (LPS) between LPS from virulent and avirulent *C. burnetii* (vLPS and avLPS, respectively) determine the pathogenic properties of *C. burnetii* (Lukacova et al., 2008; Toman et al., 2009; Toman and Vadovič, 2011; Barry et al., 2012).

To survive in macrophages, *C. burnetii* inhibits phagolysosome biogenesis (Ghigo et al., 2002; Barry et al., 2012) and induces cytoskeleton rearrangement of macrophages (Meconi et al., 1998; Honstettre et al., 2004). It has been demonstrated that LPS is the principal actor of the survival mechanism of *C. burnetii* (Meconi et al., 1998; Honstettre et al., 2004; Barry et al., 2012). vLPS stimulates morphologic changes characterized by an intense and transient membrane rearrangement of F-actin leading to protrusions and polarized projections, whereas avLPS does not induce any modification of the cell cytoskeleton morphology (Meconi et al., 1998; Honstettre et al., 2004). In addition, *C. burnetii* targeting to degradative compartments also involves an antagonistic engagement of Toll-like receptor (TLR)-4 by vLPS, lack of p38α-MAPK-driven phosphorylation, and block in recruitment of the HOPS (homotypic fusion and protein-sorting complex) component Vps41 to vLPS-containing vesicles (Barry et al., 2012).

In response to LPS stimulation, TLR-signaling initiates distinct innate immune defensive programs, such as the maturation of phagosomes (Blander and Medzhitov, 2004, 2006). This process involves crosstalk between mitogen-activated protein kinase (MAPK) signaling and components of the vesicular trafficking machinery (Blander and Medzhitov, 2006; Symons et al., 2006; Fontana and Vance, 2011). TLR-4 is involved in the recognition of Gram-negative bacteria such as *E. coli* through recognition of prototypic LPS. TLR-2 interacts with Gram-positive bacteria following interaction with lipoproteins, proteoglycans or lipopeptides. However, several studies have highlighted that LPS recognition is not restricted to TLR-4. Indeed, TLR-2 is able to recognize the LPS from *Porphyromonas gingivalis* (Medzhitov, 2001; Underhill, 2004). Recent studies have highlighted that TLR-2 is required along with TLR-4 for the response to bacterial LPS (Good et al., 2012); this response involves a physical interaction between TLR-2 and TLR-4 (Lee et al., 2004; Good et al., 2012). Much remains to be learned regarding the molecular basis underlying the crosstalk between the LPS variants and TLRs.

In this study, we investigated the mechanism leading to the absence of activation of the p38α-MAPK despite TLR-4 engagement by *C. burnetii* vLPS. We found that the association between TLR-2 and TLR-4 is required to activate p38α-MAPK and was disrupted by the vLPS. The disruption of TLR-2 and TLR-4 association by vLPS was induced by the re-organization of the macrophage cytoskeleton. Interestingly, the block of the actin cytoskeleton re-organization inhibited the disruption of association TLR-2/TLR-4 by pathogenic *C. burnetii* and allowed the p38α-MAPK activation by *C. burnetii* LPS. We elucidated an unexpected mechanism allowing pathogenic *C. burnetii* to avoid activating macrophages.

## Results

### vLPS disrupts TLR-4 and TLR-2 association at the BMDMs membrane surface

**Figure 1:**
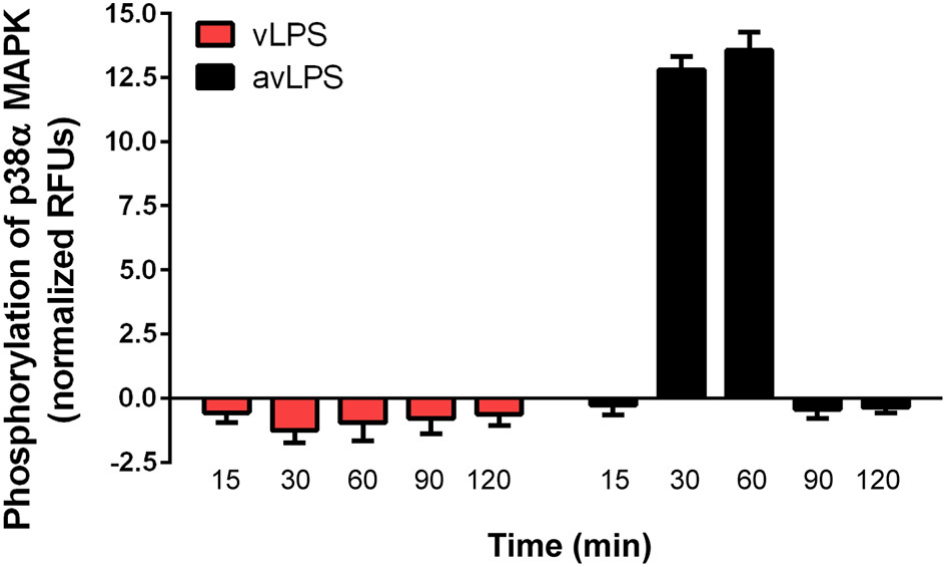
**Activation of p38α MAPKs**. BMDMs from wild type mice, were challenged with *C. burnetii* vLPS and avLPS (1 μg/ml) for different periods (up to 120 min). The phosphorylation of p38α MAPK was determined using phospho-p38α MAPK cell-based ELISA. The results are expressed as normalized RFU and represent the mean ± SD (*n* = 3).

**Figure 2:**
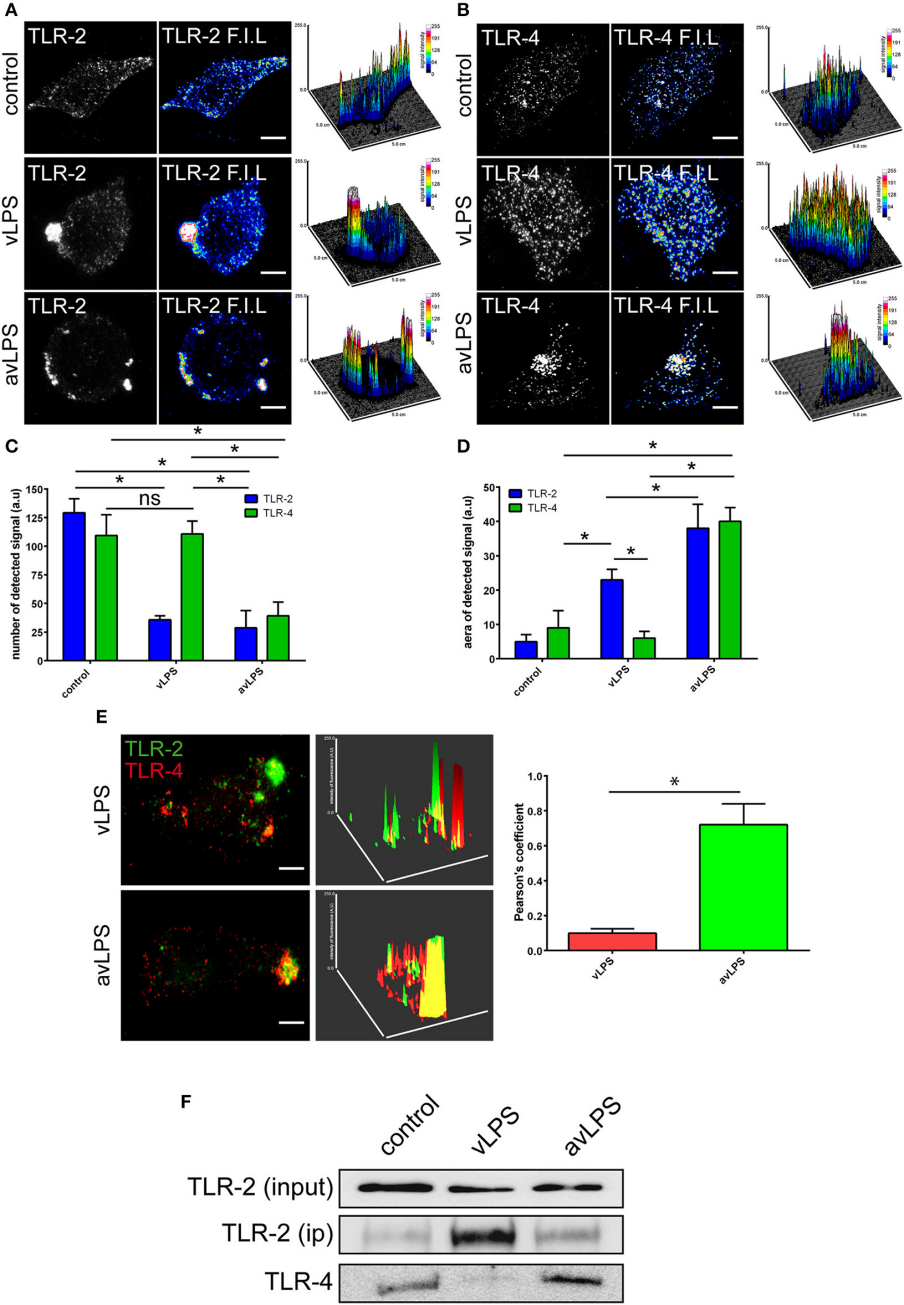
**TLR-2 and TLR-4 distribution and colocalization**. BMDMs from wild type mice were challenged for 5 min with *C. burnetii* LPS (1 μg/ml). The distribution of **(A)** TLR-2 and **(B)** TLR-4 at the BMDMs surface was determined by confocal microscopy. The scale bar indicates 5 μm. The number of TLRs signal detected **(C)** and the area **(D)** were quantified using ImageJ software. The results are expressed as the mean ± SD (*n* = 3, ^*^p < 0.05). **(E)** The colocalization of TLR-2 with TLR-4 was determined using confocal microscopy. The colocalization of TLR-2 with TLR-4 was quantified using ImageJ software. The results are expressed as the mean ± SD (*n* = 3, ^*^p < 0.05). The scale bar indicates 5 μm. **(F)** BMDMs in non-starved conditions were either left untreated or treated with vLPS or avLPS (1 μg/ml) for 5 min, then TLR-2 was immunoprecipitated and coimmunoprecipitated with TLR-4 was visualized by immunoblotting. The blot shown is representative of three experiments.

In wild-type BMDMs, vLPS was unable to induce the activation of p38α MAPK (<0 RFUs), in contrast to the avLPS (13.5 RFUs at 30 min) (Figure 1) as previously described (Barry et al., 2012). These data confirm the previous finding that the recognition of vLPS by TLRs is required to block p38α MAPK activation (Barry et al., 2012). We decided to deepen our analysis by investigating the distribution of TLR-4 and TLR-2 at the membrane surface of BMDMs challenged with *C. burnetii* LPSs (Figure 2). In control BMDMs, we observed a large number of TLR-2 and TLR-4 fluorescent small dots (129.2 ± 12.3 and 109.5 ± 18 a.u, respectively) with a dispersed distribution at the macrophage surface (Figures 2A–C) with a reduced area (5 ± 2 and 9 ± 5 a.u, respectively) (Figures 2A,B,D). In macrophages treated with the avLPS, we found a significant decrease in TLR-2 (4.5-fold) and TLR-4 (2.8-fold) dots compared to the control (Figures 2A–C) at the macrophage surface. The decreased dot number is associated with an increase in dot area (Figures 2A,B,D). Indeed the TLR-2 and TLR-4 dot area increased significantly 7.6-fold and 4.4 fold, respectively, compared to the control (Figures 2A,B,D). vLPS induced a significant a decrease of 3.6-fold of the small dots numbers present at cell membrane (Figures 2A–C) associated with a significant reorganization of TLR-2 small dots in large patch compared to control (23 ± 3 a.u vs. 5 ± 2) (Figures 2A,B,D). Interestingly, vLPS does not affect the distribution or the size of TLR-4 fluorescent dots at the BMDMs membrane surface. Then, we assessed the co-localization of TLR-4 with TLR-2 in BMDMs treated with *C. burnetii* LPSs (Figures 2E). In macrophages challenged with avLPS we find a strong co-localization of TLR-4 with TLR-2 (Pearson's coefficient 0.72 ± 0.12) whereas in presence of vLPS we found that TLR2 did not co-localize with TLR-4 (Pearson's coefficient 0.1 ± 0.02). Next, we investigated, by co-immunoprecipitation, the association of TLR-2 with TLR-4 in BMDMs either challenged or not challenged with vLPS or avLPS (Figure 2F). We observed that TLR-2 and TLR-4 co-immunopreciptated in the BMDMs control as well in BMDMs challenged with avLPS. In contrast, TLR-2 and TLR-4 did not co-immunoprecipitate in macrophages treated with vLPS. Taken together this data suggests that vLPS disrupts the membrane distribution of TLR-2 and TLR-4.

### Cytoskeleton re-organization induced by vLPS disrupts p38α MAPK activation through TLRs

**Figure 3:**
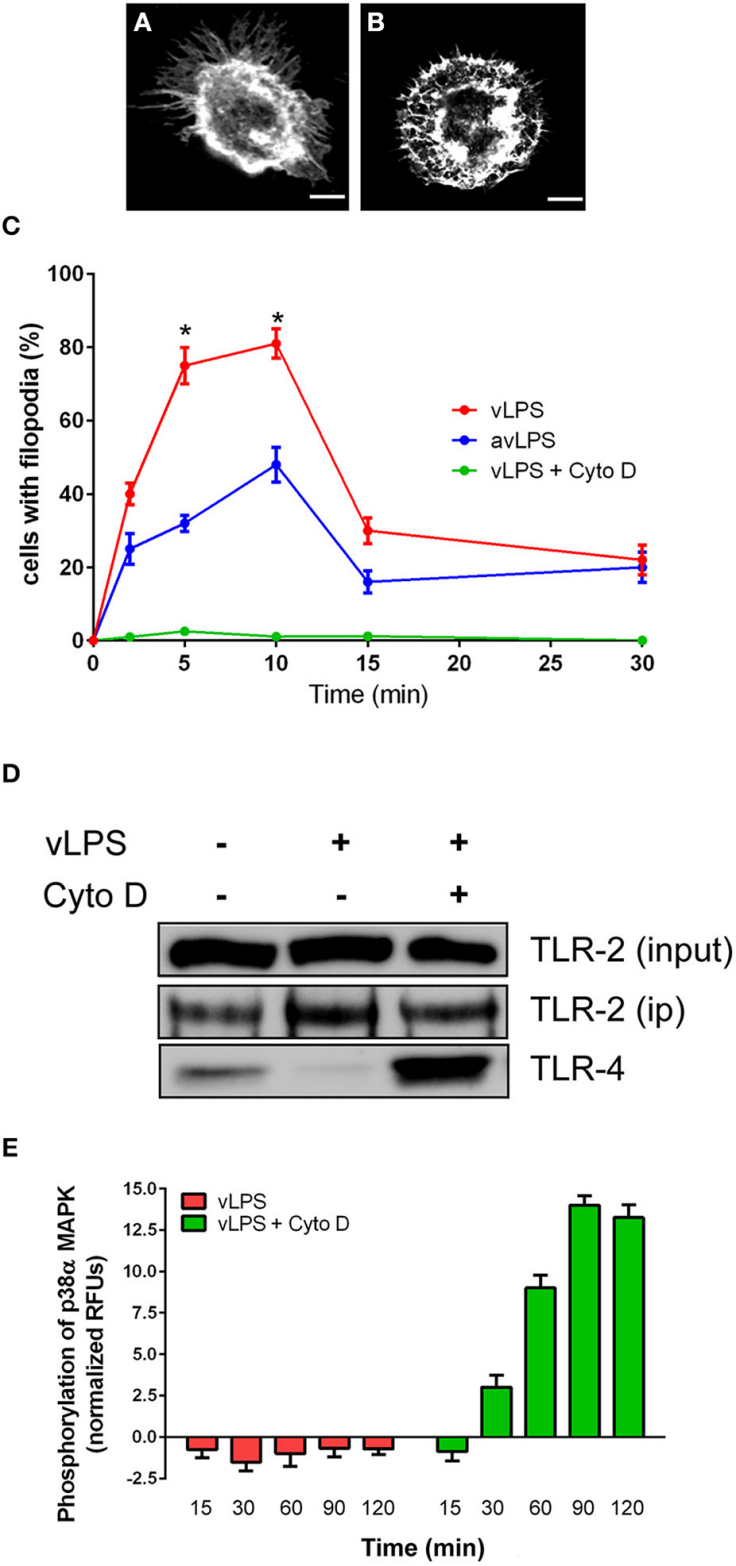
**Cytoskeleton remodeling induced by *C. burnetii* LPS impairs TLRs signaling**. BMDMs were challenged with **(A)** vLPSs or **(B)** avLPS at 1 μg/ml for 5 min, then F-actin was labeled with phalloidin alexa-488. Macrophages were examined by confocal microscopy. Representative cells are shown, the scale bar indicates 5 μm. **(C)** The percentage of BMDMs showing filopodia was evaluated. For some experiments macrophages were treated with cytochalasin-D. The results are expressed as the mean ± SD (*n* = 3 ^*^p < 0.05). **(D)** BMDMs in non-starved conditions were either left untreated or treated with vLPS (1 μg/ml) for 5 min in presence or not of cytochalasin-D, then TLR-2 was immunoprecipitated and coimmunoprecipitated with TLR-4 was visualized by immunoblotting. The blot shown is representative of three experiments. **(E)** BMDMs from wild type mice were challenged with vLPS (1 μg/ml) for different periods (min) in presence or not of cytochalasin-D, and the phosphorylation of p38α MAPK was determined using phospho-p38α MAPK cell-based ELISA. The results are expressed as normalized RFU and represent the mean ± SD (*n* = 3).

vLPS is known to induce cytoskeleton reorganization (Meconi et al., 1998; Honstettre et al., 2004). We postulated that this reorganization could influence the TLR-2 and TLR-4 distribution observed in BMDMs challenged with vLPS. We have evaluated the capacity of *C. burnetii* LPS to induce cytoskeleton reorganization. We observed, as previously described, that vLPS induced a dramatic reorganization of the BMDMs cytoskeleton (Figure 3A), whereas avLPS did not (Figure 3B) (Meconi et al., 1998; Honstettre et al., 2004). vLPS induced macrophage spreading and the formation of polarized filopodia and lamellipodia. F-actin was concentrated beneath filopodia and lamellipodia and as spots in cytoplasmic areas (Figure 3A). In contrast, avLPS had a slight effect on F-actin organization (Figure 3B). After 10 min of stimulation with vLPS, 81 ± 4% of macrophages exhibited filopodia, and the percentage of macrophages with filopodia decreased thereafter (Figure 3C). Only 35% of cells treated with avLPS exhibited filopodia (Figure 3C). Next, we have investigated if the block of the cytoskeleton re-organization could rescue the association between TLR-4 and TLR-2. We inhibited cytoskeleton re-organization using cytochalasin-D (Figure 3C). We revealed that in presence of cytochalasin-D, TLR-2 and TLR-4 co-immunoprecipitate in contrast to the experimental condition without the inhibitor (Figure 3D). Finally, we found that the inhibition of the cytoskeleton reorganization by cytochalasin-D recovers activation of p38α MAPK via vLPS (14.1 ± 0.56 RFUs after 90 min), whereas in the absence of cytochalasin-D, p38α MAPK is not activated (Figure 3E).

## Discussion

*C. burnetii*, the bacteria that causes Q fever, has evolved several strategies to survive in macrophages. One of these strategies is to avoid being targeted to the degradative compartments of immune cells. To do that *C. burnetii*, through its vLPS, blurs its own recognition by TLR receptors in order to interfere with the transduction of the signal (Barry et al., 2012). The consequences of such strategies are a deficiency of p38α-MAPK-driven phosphorylation and a block in recruitment of the homotypic fusion. In addition, several years ago it was shown that *C. burnetii* interferes with the cytoskeleton, and this inference is crucial for its survival in macrophages (Meconi et al., 1998; Honstettre et al., 2004). We have investigated if the dramatic cystoskeleton re-organization induced by *C. burnetii* could explain the decrease of p38a MAPK signaling. In macrophages challenged with *C. burnetii* avLPS we observed a phosphorylation of the p38α MAPK. In contrast, p38α MAPK is not activated in macrophages challenged with vLPS has previously described (Barry et al., 2012). Because it is known that TLR-2 is required along with TLR-4 for the response to bacterial LPS (Good et al., 2012), through a physical interaction between TLR-2 and TLR-4 (Lee et al., 2004; Good et al., 2012) we have analyzed the distribution of TLR-2 and TLR-4 at the surface of macrophages via confocal microscopy. We have observed that C*. burnetii* vLPS induces a strong reorganization of the TLR-2 and TLR4 at the membrane. This redistribution hampers the colocalization between TLR-2 and TLR-4, in contrast to what is observed in macrophages challenged by avLPS. In addition, the co-immunopreciptation experiments highlight a physical link between TLRs in cells challenged with avLPS, whereas in cells treated with vLPS this is not found. Finally, we postulated that the TLR distribution was linked to the cytoskeleton re-organization induced by *C. burnetii* vLPS. Interestingly, the inhibition of the cytoskeleton reorganization by cytochalasin D allowed for the activation of p38α MAPK by vLPS. We observed also that in this condition TLR-4 co-immunoprecipitated with TLR-2. A possible mechanism to explain the role of the vLPS in the default of activation of p38α MAPK is that vLPS through the induction of the cytoskeleton remodeling, induces a relative dispersion and redistribution of TLR-2, TLR-4 receptors at outer membrane level, in such a way that TLR-2 and TLR-4 are not able to signal via p38a MAPK. Moreover, it has been already reported that a crosstalk between TLRs signaling and G-protein coupled receptors, such as Rho-GTPase and Rnd proteins, exists and could lead to a dramatic cytoskeleton rearrangement (Ruse and Knaus, 2006). Previously, we have demonstrated that *C. burnetii* vLPS interferes with phagosome maturation by inhibiting the activation of p38α MAPK (Barry et al., 2012), here we deepened the previous study by demonstrating that *C. burnetii*, through it vLPS blurs the TLR-2 and TLR-4 signaling through dramatic cytoskeleton reorganization and redistribution of TLR-2 and TLR-4 at the macrophage cells surface.

## Materials and methods

### Ethics statement

All animal experiments were conducted according to the Guiding Principles of Animal Care and Use defined by the Ethics Committee for Animal Experimentation (N°14 designated by the National Study Committee on the Ethics of Animal Experimentation) according to the rules of Decree N°87-848 as of October 19, 1987. All of the animal experiments conducted in this study were also approved by the Ethics Committee for Animal Experimentation (N°14 from the National Study Committee on the Ethics of Animal Experimentation) where the experiments were performed (Faculty of Medicine, Marseille, experimentation permit number to Eric Ghigo 10-300122013).

### Antibodies and fluorescent compounds

**A**ntibodies specific for TLR-2 and TLR-4 were purchased from the BD Bioscience. Cytochalasin D was purchased from Sigma-Aldrich. Secondary antibodies and phalloidin alexa-448 were purchased from Invitrogen

### LPS preparations

LPS from virulent (vLPS) and avirulent (avLPS) *C. burnetii* (Barry et al., 2012) were isolated from *C. burnetii* RSA 493 (clone 7) and RSA 439 (clone 4), as previously described (Skultety et al., 1996; Toman and Skultety, 1996). The quality of the LPS preparation was confirmed using silver staining and compositional GC-MS (Toman et al., 2009).

### Cell culture

Bone marrow-derived macrophages (BMDMs) were generated from 6- to 8-week-old C57BL/6 mice, as previously described (Ren et al., 2005; Cook et al., 2007; Trouplin et al., 2013). BMDM were grown in DMEM supplemented with 10% fetal calf serum, 2 mM L-glutamine, 100 IU/ml penicillin, and 100 μg/ml streptomycin at 37°C in 5% CO_2_. For some experiments macrophages were challenged with 1 μg/ml of *C. burnetii* LPS.

### Confocal microscopy

Cells were fixed with 3% paraformaldehyde in phosphate-buffered saline (PBS pH 7.4) and prepared for immunofluorescence labeling, as previously described (Forestier et al., 1999; Chu and Ng, 2004; Ghigo et al., 2010). Coverslips were mounted in Mowiol, and the cells were imaged using an inverted Leica TCS SPE confocal laser-scanning microscope (Leica, Heidelberg, Germany). Image acquisition was performed using the Leica Confocal software. The collected images were processed using Adobe Photoshop CS5 software. The cells were evaluated as follows: twenty-five fields containing at least three cells per field were examined for each experimental condition; in total, approximately 100 cells were examined per experimental condition, as described elsewhere (Barry et al., 2012). TLR distribution at the cell surface and colocalization analyses were performed using ImageJ software (http://rsb.info.nih.gov/ij) (Bolte and Cordelieres, 2006; Barr et al., 2008). In certain experiments, morphological changes in BMDMs challenged or not challenged with *C. burnetii* LPS (1 μg/ml) were evaluated as previously described (Honstettre et al., 2004).

### P38α MAPK phosphorylation assay

The phosphorylation of p38 was assessed using phospho-p38 MAPK cell-based ELISA (R&D Systems) (Boucherit et al., 2012) following the manufacturer recommendations.

### Immunoprecipitation

BMDMs were treated with or without LPSs (1 μg/ml) for 30 min and then lysed with 1% Triton X-100 in a buffer consisting of 10 mM Tris-HCl pH 7.4, 150 mM NaCl, and 1 mM EDTA pH 8.0. TLR-4 (bdbiosciences) was immunoprecipitated via overnight incubation of the total protein with the anti-TLR-2 antibody (bdbiosciences) followed by incubation with protein ASepharose beads (Roche). The immunoprecipitated pellets were washed and analyzed via immunoblotting on 6% polyacrylamide gels using anti-TLR-2 and anti-TLR-4 antibodies. The detection of TLR-2 from the input sample was performed using 50 μg of protein. The immunoblots were visualized using an LAS 4000 camera system (GE Healthcare) or an Amersham Biosciences revelator. In some experiments, macrophages were treated with 1 μg/ml of cytochalasin D (Sigma–Aldrich) as previously described (Meconi et al., 1998; Honstettre et al., 2004).

### Statistical analysis

The results are expressed as means ± SD and were analyzed using the non-parametric Mann–Whitney *U*-test. Differences were considered significant at *p* < 0.05.

### Conflict of interest statement

The authors declare that the research was conducted in the absence of any commercial or financial relationships that could be construed as a potential conflict of interest.
